# Beyond Clavulanic Acid biosynthesis: Exploring the broad regulatory impact of BldD in *Streptomyces clavuligerus* ATCC 27064

**DOI:** 10.1371/journal.pone.0347564

**Published:** 2026-04-22

**Authors:** Luisa F. Patiño, Carlos Caicedo-Montoya, Rigoberto Ríos-Estepa

**Affiliations:** 1 Grupo de Bioprocesos, Departamento de Ingeniería Química, Universidad de Antioquia UdeA, Medellín, Colombia; 2 Departamento de Biociencias, Universidad Nacional de Colombia Sede Medellín, Medellín, Colombia; 3 Grupo de Simulación, Diseño, Control y Optimización de Procesos (SIDCOP), Departamento de Ingeniería Química, Universidad de Antioquia UdeA, Medellín, Colombia; Friedrich Schiller University, GERMANY

## Abstract

BldD is a global regulator of morphological development in *Streptomyces*, usually acting as a repressor of aerial mycelium formation. However, its role in metabolism and secondary biosynthesis in the wild-type strain *Streptomyces clavuligerus* remains poorly understood. In this study, we constructed a *S. clavuligerus* strain overexpressing *bldD* and compared it with the empty vector control strain during fermentation in soy protein isolate medium. For this, transcriptomic analyses were performed at 24 h and 72 h. We observed that BldD overexpression reduced CA production and repressed genes involved in cephamycin C biosynthesis, including *lat* and the pathway regulator *ccaR*. In contrast, genes associated with terpene biosynthesis, phage tail-like particles (CIS), and aerial mycelium formation (*bldN, bldM, chaplins*) were upregulated. These changes suggest that BldD may directly and indirectly redirects metabolic flux away from β-lactam biosynthesis while promoting morphological differentiation, alternative defense systems and a potential link with GlcNAc metabolism. By repressing β-lactam pathways and enhancing CIS and terpene gene expression, BldD shifts cellular priorities toward morphological development and ecological defense, underscoring species-specific differences from the well-studied model *Streptomyces* *coelicolor*. These results greatly contribute to the understanding of the regulatory mechanisms involved in CA biosynthesis.

## Introduction

Members of the genus *Streptomyces* are filamentous soil bacteria with a complex, multicellular life cycle. This cycle begins when spores germinate to form a vegetative mycelium that grows actively under nutrient rich conditions. When nutrients become limited, the vegetative hyphae differentiate into aerial filaments that later develop into chains of spores, enabling the organism’s dispersion [1,2].

The transition between these developmental stages is governed by a hierarchical regulatory network known as the *bld* (for *bald*) genes, whose mutations result in the loss of aerial hyphae formation [1,3]. The products of the *bld* cascade coordinate the production of surface-active molecules that lower water surface tension and allow the hyphae to breach the air medium interface [1]. Within this cascade, the transcriptional regulator BldD functions as a master node controlling morphological differentiation, acting mainly as a repressor of genes involved in developmental processes [4].

In *S. coelicolor*, BldD directly represses the transcription of the sigma factor genes *bldN* and *whiG*, both essential for distinct stages of aerial mycelium formation and sporulation [4,5]. Once BldD-mediated repression is relieved, σ^BldN^ promotes the expression of *bldM*, a response regulator required for the formation of aerial structures [2, 5]. In parallel, σ^WhiG^ activates genes such as *whiH* and *whiI*, driving spore differentiation [6]. This complex interplay illustrates how development in *Streptomyces* is tightly connected to global regulatory networks responding to environmental and nutritional cues.

Beyond morphogenesis, *bld* genes also affect secondary metabolism. Their deletion or overexpression can alter the biosynthesis of antibiotics, pigments, and extracellular enzymes, underscoring their broad regulatory influence [2,5]. For instance, *bldD* deletion in *S. roseosporus* reduces daptomycin production while accelerating sporulation [5]. Such interconnections between differentiation and specialized metabolite production are a hallmark of the genus.

BldD regulation also extends to horizontally acquired gene clusters associated with ecological interactions and defense. Notably, the expression of contractile injection systems (CIS), phage tail-like nanostructures found in *S. lividans* and *S. coelicolor*, depends on developmental regulators such as *bldA* [7,8]. These structures resemble bacteriophage tails and mediate competitive interactions between microorganisms, possibly by delivering toxic or effector proteins into rival species.

While BldD has been extensively studied in the model organism *S. coelicolor*, its role in the industrial strain *Streptomyces clavuligerus* remains unclear. *S. clavuligerus* is notable for producing the β-lactam antibiotic cephamycin C and the β-lactamase inhibitor clavulanic acid (CA), which are co-regulated by the transcriptional activator CcaR [9,10]. However, potential regulatory connections between BldD and the CA biosynthetic pathway have not been experimentally addressed.

In this study, we investigate the global effects of *bldD* overexpression in *S. clavuligerus* ATCC 27064, focusing on its influence on morphology, clavulanic acid production, and the transcriptomic landscape during fermentation. Our findings provide new insights into the multifaceted regulatory role of BldD in coordinating development, secondary metabolism, and ecological adaptation in *Streptomyces*.

## Materials and methods

### Bacterial strains, plasmids, and culture conditions

The bacterial strains, plasmids, and media compositions used in this work are summarized in Supplementary S1 and S2 Tables in S1 File. Detailed information about reagents and cloning enzymes is also provided in the supplementary file. *E. coli* DSM11539 was cultivated in LB liquid medium at 37 °C with shaking (250 rpm) for 16 h, and maintained on LB-agar plates for routine use. Glycerol (20%, v/v) stocks were stored at –80 °C for long-term preservation. *E. coli* cells were preserved on agar plates, whereas for long term storage 20% glycerol stocks were prepared and kept at −80°C.

Spores of *S. clavuligerus* were obtained by culturing the strain on GYM agar plates (28 °C, 10–15 days). Spores were harvested in sterile water, filtered through cotton wool, diluted to ≈10⁹ CFU mL ⁻ ¹, and stored in 20% glycerol at –80 °C. Spores of *S. clavuligerus* wild type and related recombinant strain were grown in suspension for 36 h (OD_600_ of 0.7) in TSB at 28°C and 220 rpm. Mycelium cultures were stored as 20% glycerol and stocked at −80ºC.

A seed medium was used for *S. clavuligerus* cultivation at pH 6.8. For CA production the soy protein isolate (ISP) medium was used [11]. The pre-culture medium had the same composition as that of the culture medium, except for glycerol concentration which was adjusted to 15 gL^-1^. All *S. clavuligerus* cultures were carried out in 250-baffled Erlenmeyer flasks containing 50 mL of medium. Pre-culture flasks were inoculated with seed medium (10% v/v), and the cultivation medium was inoculated with 10% v/v of pre-culture medium. Cultures were incubated for 120 h, at 220 rpm and 28 ^◦^C. All experiments were performed in triplicate.

### Construction of recombinant plasmids and transformation

To overexpress the *bldD* gene in *S. clavuligerus*, the *bldD* open reading frame was amplified by PCR, inserted into the TOPO-TA vector, and subsequently subcloned into pIB139 via *Nde*I and *Xba*I sites, generating pIBLD. The insertion of *bldD* into the plasmid was confirmed by 1% (w/v) agarose electrophoresis gel and sequencing. The plasmids (pIBLD and pIB139) were propagated in *E. coli* DH5α cells and subsequently introduced into the *E. coli* DSM 11539 host. Transformation of *S. clavuligerus* was achieved through a PEG-mediated protoplast [11]. The protoplasts were obtained from cultures grown at 30 °C in YEMEG medium under previously described conditions [11]. The recombinant strains were termed as *S. clavuligerus*/pIBLD and *S. clavuligerus*/pIB139, respectively.

The presence of the plasmid pIBLD in *S. clavuligerus* was confirmed by PCR using the reverse primer and a forward primer, designed from an inner region of the promoter ermE*. To confirm insertion of pIB139 in *S. clavuligerus* the primers were designed from the apramycin resistance gene, *aac(3)* IV (S3 Table in S1 File). The control strain (*S. clavuligerus*/pIB139 having vectors without any gene inserted) was constructed to determine the potential effect of pIB139 on CA yield for a recombinant strain and to evaluate the effect of the cloned gene on CA production without considering effects caused by the presence of pIB139.

### Clavulanic acid fermentation and analytical techniques

Three independent biological replicates of the control and recombinant *S. clavuligerus* strains were cultured under identical conditions. For sampling, aliquots of 2 mL were taken from each flask every 24 hours during the fermentation process. For CA quantification, cell culture samples were centrifuged at 14,000 × g for 10 min at 4°C and filtered (0.22 µm). CA concentrations were quantified by HPLC (Agilent 1200 series, DAD detector, 312 nm) on a ZORBAX Eclipse XDB-C18 (4.6 × 150 mm, 5 µm) column. The mobile phase consisted of 50 mM KH₂PO₄ (pH 3.2) and 6% methanol (v/v) at 0.7 mL min ⁻ ¹. CA was imidazole-derivatized at a ratio of 1:3; the reaction was kept at 28°C for 15 min [12].

### RNA extraction and RNA sequencing (RNA-Seq) analysis

RNA was extracted in duplicate for *S. clavuligerus* with the recombinant plasmid pIBLD and for the control strain at 24 and 72 hours of cultivation, when the conditions evaluated were minimum and maximum for CA production, respectively. 2 mL of the sample was centrifuged at 10,000 g for 15 min at 4°C; the supernatant was stored for downstream analysis; cell pellets were resuspended in 800 μL of Monarch RNA protector 1X (New England Biolabs) and immediately stored at −80°C for subsequent RNA extraction. Total RNA was isolated from frozen mycelia using the RNeasy Plus Universal Mini Kit (Qiagen, Germany) according to the manufacturer’s recommendations. The RNA samples were quantified using Qubit 2.0 Fluorometer (ThermoFisher Scientific, Waltham, MA, USA) and RNA integrity was checked with 4200 TapeStation (Agilent Technologies, Palo Alto, CA, USA). Only samples with high-quality RNA (RNA integrity number ≥ 7.0) were used in the following mRNA library preparation. For library preparation and sequencing, samples were initially treated with TURBO DNase (Thermo Fisher Scientific, Waltham, MA, USA) to remove DNA contaminants. The next steps included performing rRNA depletion using QIAseq® FastSelect™ − rRNA Bacteria (Qiagen, Germantown, MD, USA), which was conducted following the manufacturer’s protocol. Strand-specific libraries were generated with the NEBNext Ultra II Directional RNA Library Prep Kit for Illumina (NEB, USA) following the supplier’s guidelines (NEB, Ipswich, MA, USA). The sequencing library was validated on the Agilent TapeStation (Agilent Technologies, Palo Alto, CA, USA), and quantified using Qubit 2.0 Fluorometer (ThermoFisher Scientific, Waltham, MA, USA) as well as by quantitative PCR (KAPA Biosystems, Wilmington, MA, USA). The sequencing libraries were multiplexed and clustered on one lane of a flowcell. After clustering, the flowcell was loaded onto the Illumina HiseqTM 2000 (Illumina, San Diego, CA, USA) according to manufacturer’s instructions. The samples were sequenced using a 2x150bp Paired-End (PE) configuration and ~350M PE read.

### Data processing

Quality control of the FASTQ format raw data was checked using FastQC [13], then the raw data were trimmed by removing adapter sequences and reads with low quality, using the software fastp (v0.23.4) [14]. The high-quality reads were used in subsequent alignments. The sequencing paired-end reads were mapped to the corresponding reference genome sequence (Genome accession numbers for *Streptomyces clavuligerus* ATCC 27064, NZ_CP027858.1 and NZ_CP027859.1, chromosome and megaplasmid, respectively) using Bowtie2 (Version 2.5.1) [15]. Mapped read was exported as a BAM file format using Samtools [16]. Aligned sequencing reads were counted using HTSeq-count [17] based on all annotated genomic features of the *S. clavuligerus* reference genome (NCBI genome assembly GCF_005519465.1). The DESeq2 package was used to normalize the data and identify differentially expressed genes; only genes with a *p* < 0.05 and adjusted *p*-value (*padj)* ≤ 0.05 were considered statistically significant and retained for further analysis [18]. The raw sequencing data were deposited in the SRA database of NCBI under the accession number PRJNA1125460.

### Public transcriptome datasets and comparative analyses

Public transcriptome datasets were used for comparative context: the *S. coelicolor bldD* null mutant microarray study (ArrayExpress E-MEXP-2853; processed GC-RMA files; differential expression computed with limma using the A-AFFY-85 annotation). Cross-species comparisons used reciprocal best-hit orthology (DIAMOND BLASTP) and were summarized by Fisher’s exact test (DEG overlap) and Spearman correlation of ortholog fold-changes. Because the *S. coelicolor* dataset reflects loss of *bldD* function (*bldD* knockout) whereas our dataset reflects *bldD* overexpression, fold-changes from the overexpression condition were multiplied by −1 prior to rank-based comparisons to align expected directionality under opposite perturbations.

#### Promoter motif enrichment analysis.

Promoter regions comprising 250 bp upstream of each DEG were analyzed using MEME Suite (v5.1.1) [19] to detect conserved sequence motifs. Identified motifs were subsequently compared to reference databases through TOMTOM to infer potential regulatory patterns [20] when required. The highest-scoring motif was selected for subsequent analyses. To statistically assess motif enrichment, we defined an explicit background consisting of 250-bp upstream promoter regions from non-differentially expressed genes (*padj* > 0.05) at each time point. Motif enrichment in DEG promoters versus the background was tested using AME (MEME Suite), and individual motif occurrences were scanned using FIMO. For FIMO, promoters were classified as ‘hit’ if at least one match passed the selected q-value (or *p*-value) threshold, and enrichment was assessed by Fisher’s exact test (DEG vs non-DEG promoters). Analyses were performed separately for 24 h and 72 h.

### Statistical analysis

The experimental results were statistically analyzed using the R software (version 4.4.1) [21]. For CA results, data were evaluated by the Shapiro-Wilk normality test. Parametric data were subjected to analysis of variance (ANOVA) and Tukey’s HSD test. Significance level was set as *p* < 0.05.

## Results

### Clavulanic acid production by *S. clavuligerus*/pIBLD

**Fig 1:**
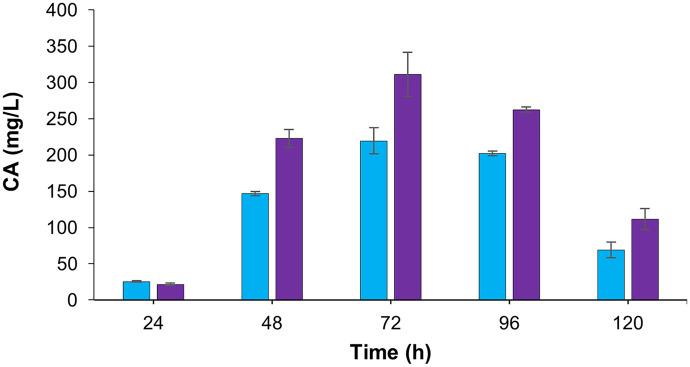
Production of CA by the recombinant strains. *S. clavuligerus*/pIB139 (purple), and *S. clavuligerus*/pIBLD (blue).

To evaluate the influence of *bldD* overexpression on CA biosynthesis, comparative fermentations of the recombinant strain (*S. clavuligerus*/pIBLD) and the control strain (*S. clavuligerus*/pIB139*)* were carried out in ISP medium. The overexpressing strain produced a maximum of 219.4 mg/L CA after 72 h, corresponding to a 1.4-fold reduction relative to the control (311 mg/L; *p* < 0.05). This decrease was consistently observed from 48 to 120 h of cultivation (Fig 1).

**Fig 2:**
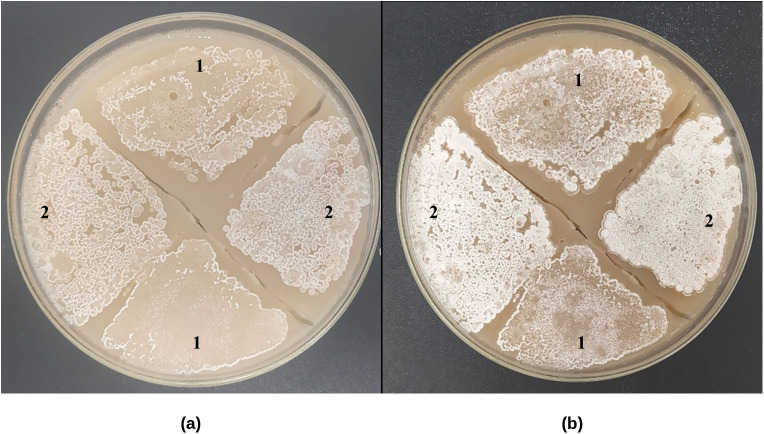
Aerial mycelium formation of *S. clavuligerus* on GYM medium with apramycin 40 µg mL^-1^ (1) *S. clavuligerus*/ pIB139. (**2**) *S. clavuligerus*/ pIBLD. **(a)** After 5 days of growth. **(b)** After 10 days of growth.

Fig 2 illustrates the effect of *bldD* overexpression on aerial mycelium formation in *S. clavuligerus*. The recombinant strain harboring pIBLD (Fig 2) exhibited abundant aerial mycelium after 5–10 days of cultivation, whereas the control strain carrying the empty vector pIB139 (Fig 2) displayed only limited mycelial development. These results show that *bldD* plays a role in the initiation of aerial growth under the tested conditions.

### RNA sequencing and differential expression analysis for *S. clavuligerus*/pIBLD

#### Transcriptome analysis at 24 and 72 hours of culture.

**Fig 3:**
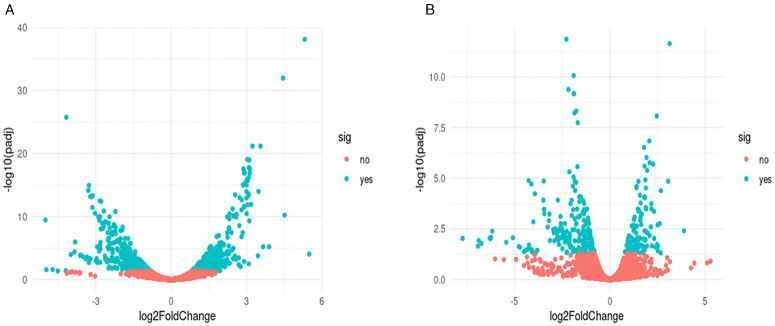
Volcano plot showing differentially expressed genes. Cyan dots indicate the significantly upregulated and downregulated genes. Pink dots indicate genes that were not significantly different in the two groups. The X-axis depicts the log2 fold-change in expression, while the Y-axis represents the statistical significance of each gene. A) 72 hours, B) 24 hours.

To explore transcriptional changes associated with *bldD* overexpression, RNA-seq comparisons were performed between the recombinant and control strains at 24 h and 72 h of growth. At 72 h, the stage of maximal CA accumulation, 372 genes showed increased expression and 366 were repressed. Most regulated genes were located on the chromosome (88% of upregulated and 63% of downregulated), while the remaining ones mapped to the megaplasmid pSCL4 (Fig 3A; S2,S3 Files). At 24 h, 122 genes were induced and 183 repressed (Fig 3B; S4 File).

Pathway enrichment (KEGG annotation) [22] showed that upregulated genes at 72 h were predominantly linked to primary metabolism, including carbohydrate, lipid, and amino acid pathways, as well as secondary metabolite biosynthesis such as terpenoids and polyketides. Conversely, genes involved in transport, sulfur metabolism, and oxidative phosphorylation were repressed, particularly those connected with cephamycin C and CA biosynthesis (S1 Fig in S1 File).

**Fig 4:**
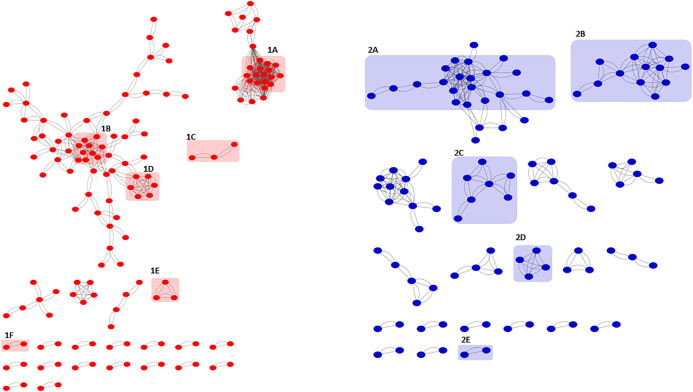
Predicted STRING networks for the DEGs at 72 h of cultivation, visualized in Cytoscape. The network in red corresponds to the up-regulated genes with some representative highlighted clusters. Cluster **1A**: Bacteriophage T4, Gp19, tail tube. **1B**: Fatty acid and lipid metabolic process. **1C**: Sesquiterpenoid and triterpenoid biosynthesis. **1D**: Organic acid metabolic process. **1F**: Cysteine and methionine metabolism and 1F: Lanthionine synthetase C-like. In blue, the network for the down-regulated genes with some representative highlighted clusters. **2A**: Penicillin and cephalosporin biosynthesis. Cluster **2B**: Sulfur metabolism. **2C**: aminopeptidase activity and whiB. **2D**: Glycine betaine transport; **2E**: Beta-lactamase and acetyltransferase domain.

**Fig 5:**
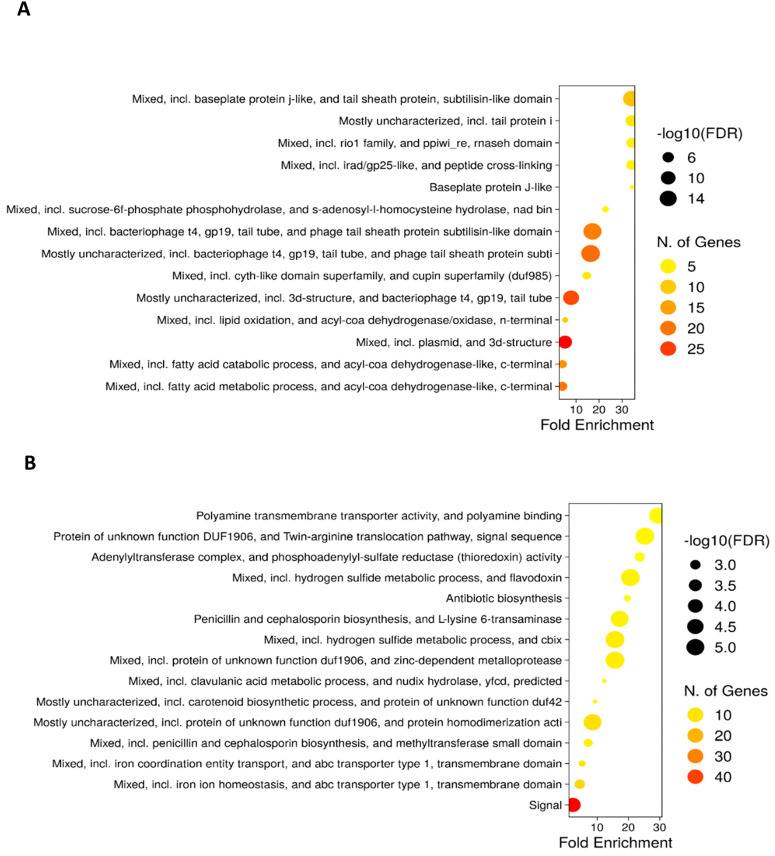
Ontology enrichment analysis (FDR <= 0.05) generated in ShinyGO v0.741 for *S. clavuligerus/*pIBLD at 72 h. **A)** Up-regulated genes. **B)** Down-regulated genes.

Protein–protein interaction (PPI) networks predicted with STRING [23] and visualized in Cytoscape [24] revealed significant enrichment (*p* < 1.0e^-16^): upregulated DEGs formed a network of 205 nodes and 129 edges, while downregulated DEGs formed one with 127 nodes and 93 edges (Fig 4). Functional enrichment with ShinyGO (15) further highlighted associations between metabolic and developmental processes (Fig 5).

**Fig 6:**
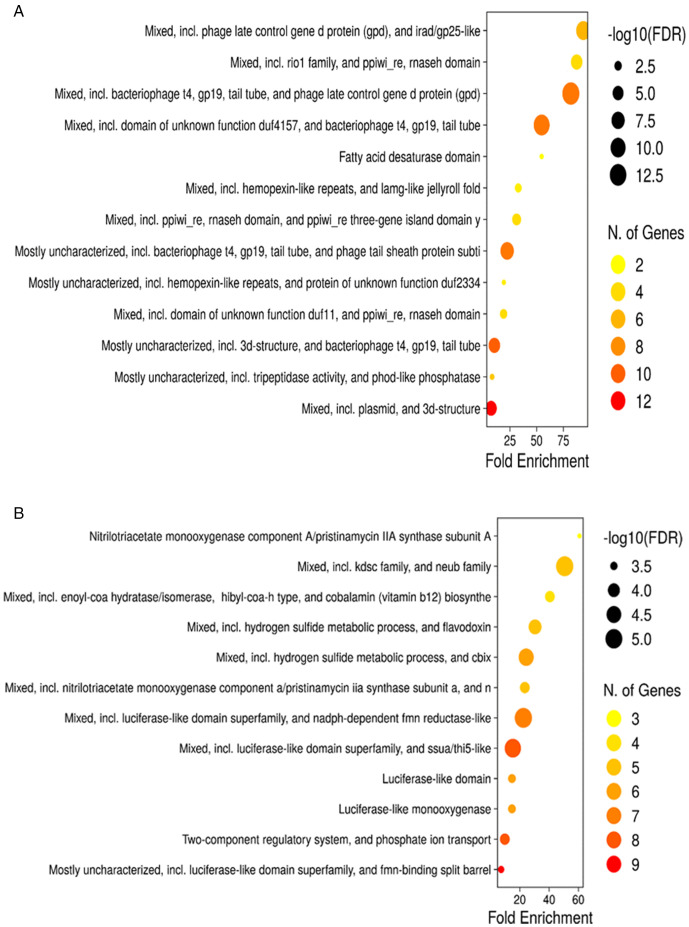
Ontology enrichment analysis (FDR<=0.05) generated in ShinyGO v0.741150 for DEGs at 24 hours of growth. **A)** Up-regulated genes. **B)** Down-regulated genes.

Conversely, at 24 hours of cultivation, most of the genes that were up-regulated were those related to phage tail sheath proteins (Fig 6A). Regarding the genes that decreased their expression, it was found that these are related to phosphate ion transport, sulfur metabolism and two-component regulatory system (Fig 6B). Below are described some of the most representative differentially overexpressed genes at 24 and 72 hours of growth.

#### Organization of phage tail related genes in *S. clavuligerus.*

**Fig 7:**
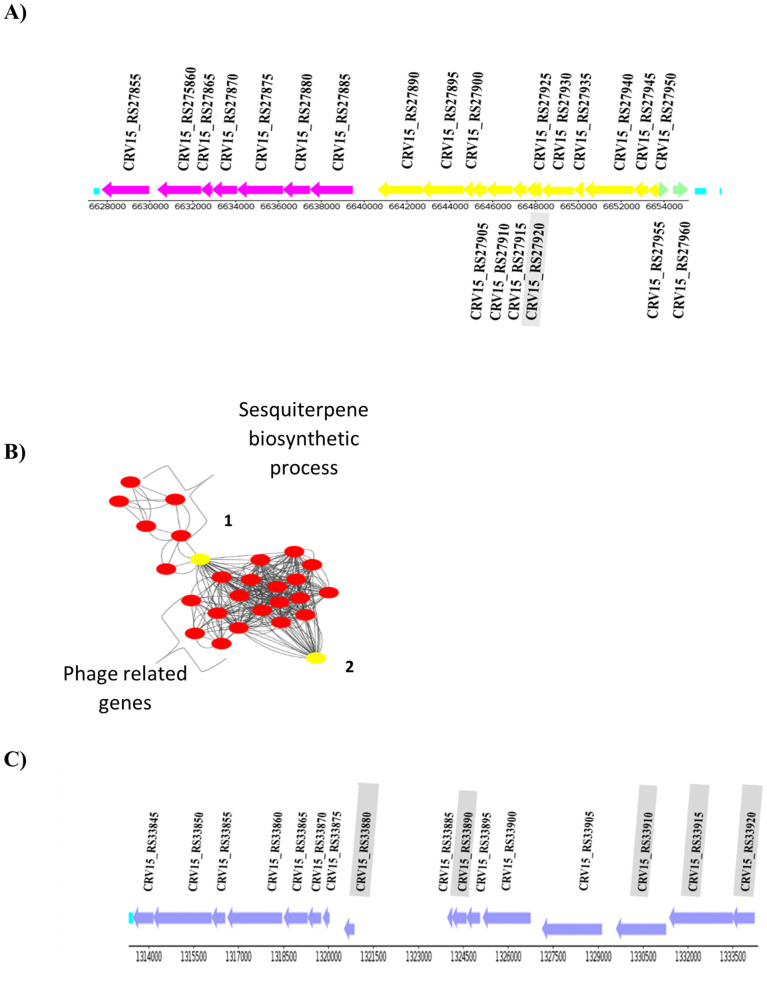
Up-regulated genes related to the Phage-like protein in *S. clavuligerus.* **A)** Phage-related genes and their potential organization in *S. clavuligerus* ATCC27064 chromosome. **B)** Potential interaction network for phage-related genes predicted using STRING DB (version 12.0). 1.Terpene synthase (CRV15_RS00255). 2 BTAD putative transcriptional regulator (CRV15_RS27960). **C)** Phage-related genes and their potential organization in *S. clavuligerus* ATCC 27064 plasmid. Genes that were not overexpressed are highlighted in grey.

CIS-related gene expression increased at both 24 h and 72 h. Early in cultivation (24 h), the overexpressed cluster was mainly plasmid-borne (Fig 7C), whereas at 72 h the chromosomal cluster showed 2–3.5-fold higher expression levels (Fig 7A). Network analysis (STRING-DB) indicated possible functional connections between CIS genes and terpene biosynthetic enzymes (score = 0.772), suggesting a metabolic or regulatory association (Fig 7B). In addition, CRV15_RS27960, encoding a SARP-family DNA-binding regulator, was predicted to interact with phage-related genes based on genomic co-occurrence and gene neighborhood, supporting its possible role in cluster regulation. Although bioinformatic analyses suggested potential functional connections, these relationships are still hypothetical and require experimental confirmation.

#### Overexpressed genes involved in different cellular processes.

*bldD* overexpression enhanced the transcription of genes related to carbohydrate and amino acid metabolism, along with putative signaling components. For example, CRV15_RS26965 and CRV15_RS21135 (S5 Table in S1 File), predicted to encode glucosamine-6-phosphate deaminase and α-N-acetylglucosaminidase, respectively (KEGG annotation), may participate in processes linking morphology and secondary metabolism in *S. clavuligerus*/pIBLD. Regarding secondary metabolism, several genes involved in the biosynthesis of terpenes and related molecules were identified (S5 Table in S1 File). As an example, the gene CRV15_RS00255, which encodes a terpene synthase, showed a 2.5-fold increase in its expression. This gene is located in the same cluster as CRV15_RS00250 and CRV15_RS00260, which encode a prenyltransferase/squalene oxidase repeat-containing protein (1.9-fold change) and a cytochrome P450 (2.5-fold change), respectively. Finally, the genes CRV15_RS02740, CRV15_RS02745 and CRV15_RS02750 increased their expression by 1.9, 2.8 and 2.1-fold, respectively. These genes are related to the biosynthesis of pyrroloquinoline quinone which could have a role in the biosynthesis of terpenes and related molecule [25].

#### Genes related to morphological development of *S. clavuligerus*/pIBLD.

Among the overexpressed genes related to morphological development, a gene that encodes the RNA polymerase sigma factor *bldN* (CRV15_RS16730), and a gene, encoding a two-component transcriptional regulator of the LuxR family (CRV15_RS09965), were identified. The latter contains an RpfG region, which is involved in signaling with the cyclic nucleotide c-di-GMP, a signaling molecule that regulates various cellular processes, including morphological development and sporulation [26]. Additionally, the gene annotated as CRV15_RS27195, which encodes the SigB/SigF/SigG family RNA polymerase associated with the sporulation process, and the gene CRV15_RS23175, encoding the chaplin protein, were highly upregulated. Regarding the chaplin protein, this type of protein is required for aerial development in *S. coelicolor*. The chaplin protein encoded by CRV15_RS23175 shows a 75.5% identity and an *E*-value of 6e^-30^ with the chaplin E protein from S. *coelicolor*. In *S. coelicolor* and other streptomycetes, chaplins act synergistically with SapB, a lantibiotic-like peptide associated with surface differentiation in nutrient-rich media [27]. In *S. clavuligerus*, SapB is encoded by the gene annotated as CRV15_RS03785, which is clustered with CRV15_RS03790 (S2 File). Both genes, responsible for producing the lantibiotic-like peptide, were overexpressed during the overexpression of *bldD***.**

#### In silico comparison with public transcriptomes.

**Fig 8:**
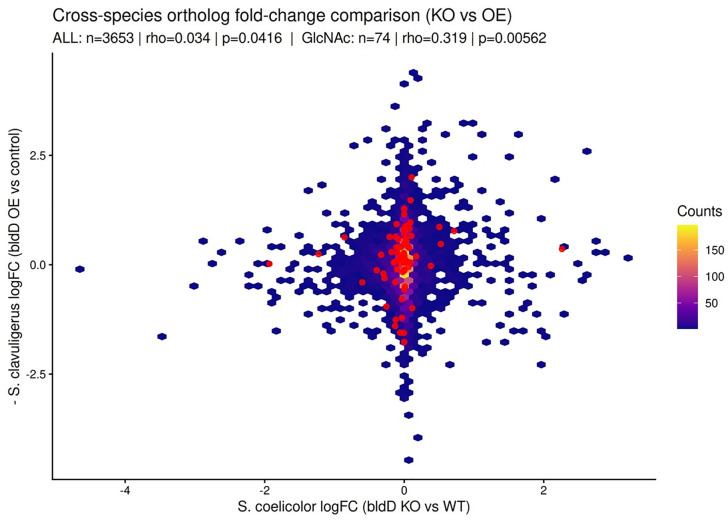
Cross-species ortholog fold-change comparison under opposite BldD perturbations. Hexbin plot of RBH orthologs (n = 3,653) comparing *S. coelicolor* *bldD* null mutant transcriptome (KO) logFC (x-axis) vs sign-inverted *S. clavuligerus* *bldD* overexpression (OE) fold-change (y-axis; − logFC). Red points highlight GlcNAc/amino-sugar orthologs (n = 74); Spearman *ρ* and *p*-values are shown for the global set and this subset.

We compared our *S. clavuligerus* *bldD* overexpression RNA-seq dataset with the published *S. coelicolor* *bldD* null mutant (knockout) transcriptome using RBH orthology (3,897 pairs). Strict DEG overlap (FDR < 0.05 in each dataset) was null (k = 0), whereas rank-based comparison across orthologs with fold-changes available in both datasets (n = 3,653) showed a statistically detectable but biologically negligible global association after accounting for opposite perturbations (*bldD* knockout vs *bldD* overexpression; Spearman ρ = 0.034, p = 0.0416, Fig 8). In an exploratory module scan, GlcNAc/amino-sugar orthologs (n = 74) showed a clearer, directionally consistent signal (Spearman *ρ* = 0.319, *p* = 0.0056, Fig 8), while refined phage/CIS orthologs showed a non-significant trend (n = 12; p = 0.14).

### Promoter motif enrichment analysis

Motif discovery with MEME identified a 41-bp AT-rich sequence enriched in the promoters of upregulated genes (E-value = 8.9e^-28^) (S2 Fig in S1 File). Although most positions were weakly conserved, a short CTCT core was consistently present and considered the functional consensus. This motif was detected in regulatory genes such as *bldN* and CRV15_RS27960. TOMTOM analysis (p = 6.38e^-03^) revealed similarity to binding sites of NagR/NagQ regulators, which control chitin and N-acetylglucosamine metabolism in Proteobacteria (S2 Fig; S5 File in S1 File).

In contrast, downregulated genes shared a GC-rich palindromic motif (E = 4.7e^-22^), with the consensus sequence 5´-TGCTCCGTCRNNNTCG-3´, identified in over 100 promoters (S2 Fig in S1 File). Importantly, this motif was found upstream of *lat* (a key gene in the cephamycin C biosynthetic cluster), as well as in genes encoding ABC/MFS transporters, TetR/AcrR-family regulators, and proteins associated with secondary metabolism and stress responses (S5 File).

Although MEME recovered a GC-rich palindromic motif among downregulated promoters, formal enrichment tests using an explicit non-DEG promoter background (AME and FIMO-based hit counting) did not support significant enrichment of this motif in downregulated genes beyond background expectations (S6 File and S7 File). In addition, scanning with the canonical BldD motif did not show preferential occurrence in the downregulated promoter set. Therefore, we treat this GC-rich motif as a candidate regulatory signature rather than evidence of direct BldD binding.

### Down-regulated genes

#### Clavulanic acid and cephamycin C.

**Fig 9:**
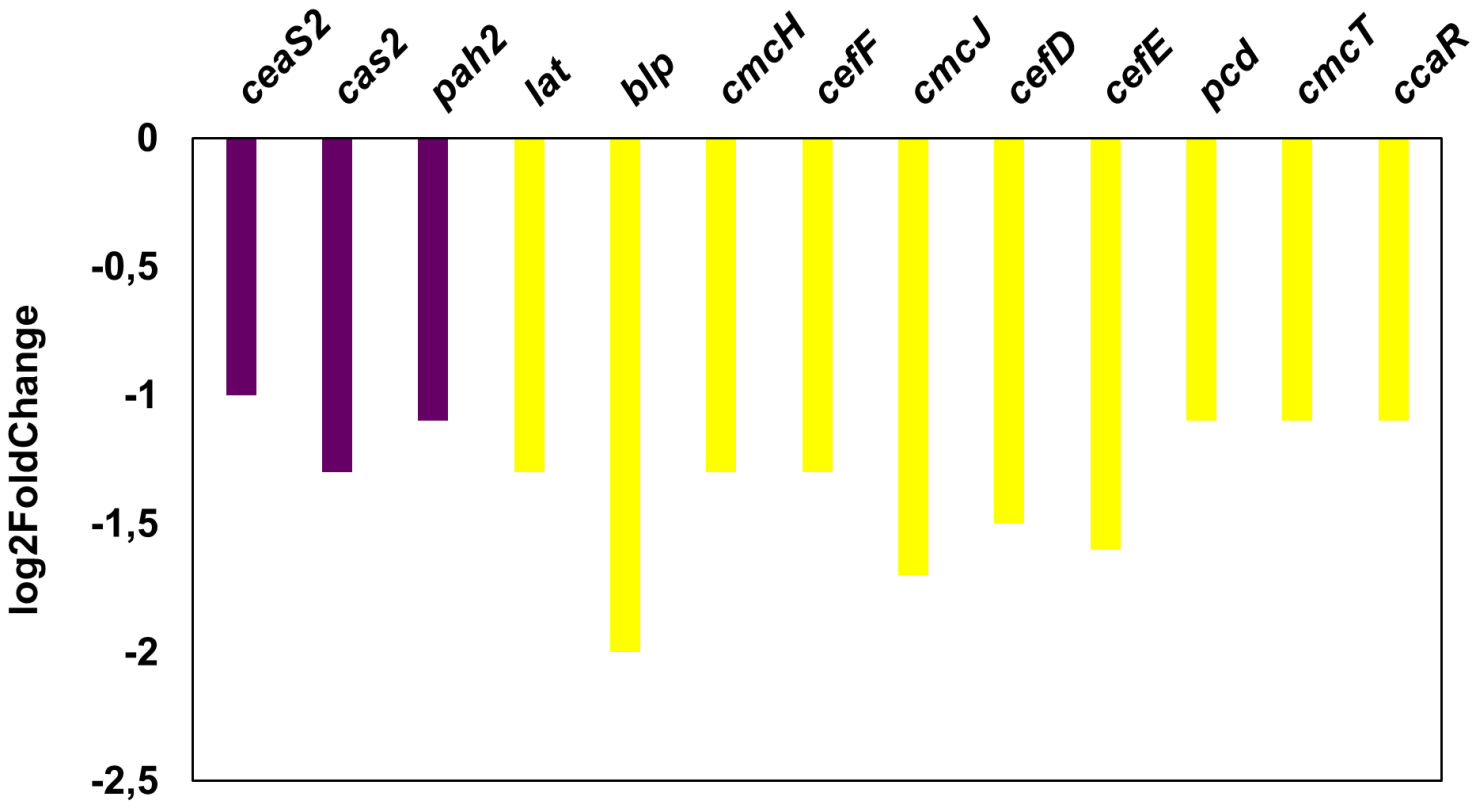
Genes from the CA and cephamycin C gene clusters that were downregulated in *S. clavuligerus*/pIBLD at 72 h. In purple, CA genes. In yellow, cephamycin C genes.

The overexpression of *bldD* in *S. clavuligerus* ATCC 27064 reduced CA production by 1.4-fold at 72 hours of culture, which is consistent with the gene expression results obtained through RNA-seq analysis. In particular, CA biosynthetic genes, namely, *ceaS2* (CRV15_RS07530), *pah2* (CRV15_RS07540), and *cas2* (CRV15_RS07545), were significantly downregulated (Fig 9). Similarly, genes within the cephamycin C cluster also showed reduced expression (Fig 9). In contrast, no significant differences were observed in the expression rates of the CA and cephamycin C clusters at 24 hours of culture, compared to the control strain.

#### Genes involved in different biological processes.

At both 24 h and 72 h, a set of genes related to cell wall structure and regulation were consistently downregulated. The peptidoglycan-binding protein gene CRV15_RS11635, together with neighboring genes encoding a hypothetical protein (CRV15_RS11640) and an ABC transporter (CRV15_RS11630), showed reduced expression (1.3–2.9-fold; S6 Table in S2 File). At 72 h, the β-lactamase inhibitor BLIP (CRV15_RS04920) also decreased (1.6-fold), while no other β-lactamase inhibitors were affected (S6 Table in S2 File).

Among the most strongly affected genes was CRV15_RS32005, encoding an AfsA-related protein, located on the plasmid alongside a methyltransferase (CRV15_RS32010) and a pyruvate-binding protein (CRV15_RS32015). All three were significantly downregulated (S7 Table in S2 File). Finally, three SigE sigma factor genes (CRV15_RS08465, CRV15_RS14420, CRV15_RS15430), which are involved in cell envelope stress responses and morphological differentiation, were also repressed (1.1–1.9-fold; S6 Table in S2 File).

## Discussion

### CIS/phage tail-like and terpene-related responses under *bldD* overexpression in *S. clavuligerus* ATCC27064

Overexpression of *bldD* stimulated CIS-related gene clusters located in both the plasmid and chromosome. Each cluster encodes distinct accessory and regulatory proteins, suggesting functional differentiation across growth phases. The plasmid-associated system may reflect functional differentiation in early competitive signaling, whereas the chromosomal one could act during later developmental transitions. Altogether, these observations indicate that *bldD* modulates adaptive and morphological responses, in agreement with findings in other *Streptomyces* species [11,12].

The concomitant upregulation of terpene biosynthetic genes suggests a coordinated response. Since terpene and lipid biosynthetic routes share common intermediates, their concurrent activation with CIS genes likely indicates a metabolic reprogramming toward ecological defense. In later growth stages, *bldD* overexpression seems to favor pathways linked to interspecies interaction instead of β-lactam synthesis [7,28].

### Links between BldD, NagR-like regulation, and cell wall metabolism

The transcriptomic results revealed increased expression of *nagB* and genes encoding enzymes involved in N-acetylglucosamine (GlcNAc) metabolism, such as α-N-acetylglucosaminidase [29]. The transcriptional pattern suggests that changes in GlcNAc utilization may connect *bldD* function with CIS activation through the action of a NagR-like regulator (CRV15_RS27960) [30]. This regulatory connection could coordinate cell wall turnover, carbon flux, and developmental progression in *S. clavuligerus.*

**Fig 10:**
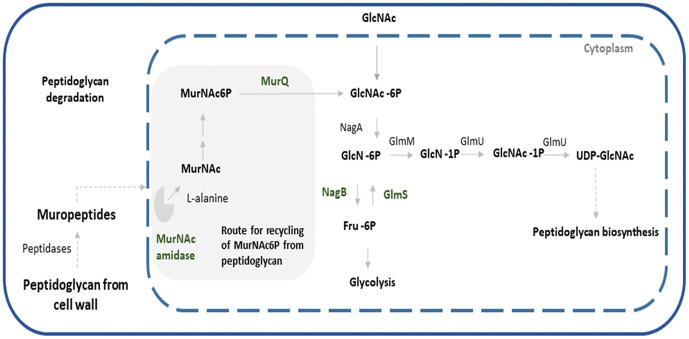
Schematic diagram of the enzymes that were overexpressed in a potential peptidoglycan biosynthesis pathway based on those in other bacteria [29,31]. In red the enzymes that were overexpressed in this work. The enzymes and substrate are described in the text.

GlcNAc links primary metabolism with cell wall formation, serving as both a nutrient and a building block for peptidoglycan synthesis [30]. In the overexpressing strain, the elevated transcription of *nagB* suggests that GlcNAc-6P may be routed toward glycolytic intermediates, while recycling through MurQ could regenerate UDP-GlcNAc for new cell wall synthesis. These routes are schematized in Fig 10, which illustrates how peptidoglycan fragments can be recycled into central metabolism or cell wall biosynthesis.

Together, these observations are consistent with a link between enhanced GlcNAc/amino-sugar metabolism, cell-wall recycling and developmental transitions, and they suggest that these metabolic cues may contribute to CIS-associated responses under *bldD* overexpression. However, because our conclusions are primarily based on transcriptomic associations, it remains difficult to distinguish which components reflect direct BldD targets versus indirect downstream effects. Notably, *bldD* overexpression altered the expression of multiple regulatory genes (including sigma factors, two-component systems and transcriptional regulators), consistent with propagation through regulatory cascades that can couple morphogenesis, metabolic allocation and ecological/defense-related programs. Regulator-focused network inference approaches, ideally integrated with direct binding validation (e.g., ChIP/EMSA), will be valuable to disentangle direct versus indirect paths linking BldD to GlcNAc utilization, CIS activation and shifts in specialized metabolism.

### BldD and its role in the expression of genes related to the morphological state of *S. clavuligerus*

In this work, *S. clavuligerus*/pIBLD exhibited increased expression of several genes related to morphological differentiation. The gene *bldN* (CRV15_RS16730), encoding an extracytoplasmic function sigma factor, was among the most induced. In *S. coelicolor,* this sigma factor is essential for the differentiation of aerial hyphae and spore chains [32]. Its transcription depends on upstream regulators such as *bldG* and *bldH,* whereas BldD negatively controls its expression. Once active, σ^BldN^ promotes *bldM* transcription, another regulator necessary for aerial growth [32,33]. Downstream of this pathway, σ^BldN^ also activates genes encoding chaplin and rodlin proteins that form the hydrophobic sheath covering aerial hyphae and spores [27]. By lowering surface tension, these proteins facilitate the emergence of aerial hyphae and promote the formation of a hydrophobic surface layer [27].

From the current study, at 72 hours of cultivation, *S. clavuligerus*/pIBLD increased the expression of *bldN* (CRV15_RS16730), *bldM* (CRV15_RS09965), and a putative chaplin E gene class (CRV15_RS23175) by 1.4-, 2.3-, and 2.1-fold, respectively. These results contrast with what has been reported for *S. coelicolor*, highlighting the species-specific regulatory roles of BldD, which vary depending on genetic and environmental contexts. Although BldD in *S. coelicolor* is reported to repress aerial mycelium formation, in *S. clavuligerus*, it may function differently through the indirect activation of developmental and regulatory genes associated with AT-rich motifs.

In *S. clavuligerus*/pIBLD, BldD likely enhances the transition from vegetative to aerial mycelium, as evidenced by the increased expression of *bldN*, *bldM*, and chaplin-related genes (Fig 2). In liquid cultures, the MII mycelium is considered analogous to the aerial mycelium found in solid cultures [34]. This mycelium is typically covered by a hydrophobic layer, which facilitates the transition to pseudo-sporulation. Additionally, an increase in aerial mycelium formation is supported by the upregulation of CRV15_RS03785 and CRV15_RS03790, genes associated with the synthesis of SapB, a small peptide with high surface activity reported in *S. coelicolor* [32]. In *S. coelicolor*, SapB contributes to aerial growth by decreasing surface tension [35]. In contrast, several *sigE* genes were transcriptionally repressed at both time points. SigE participates in a signaling network that detects alterations in the cell envelope and initiates adaptive responses in *Streptomyces* [28]. Its downregulation in the *bldD*-overexpressing strain coincided with the upregulation of developmental genes, suggesting an inverse relationship between stress signaling and morphogenesis.

### Effect of BldD overexpression on clavulanic acid production

It was observed that BldD negatively affected the transcription of genes involved in the biosynthesis of cephamycin C and CA by downregulating their expression. In *S. clavuligerus*, the transcriptional activator CcaR governs the coordinated expression of both the CA and cephamycin C biosynthetic clusters [10]. It promotes transcription of genes within these pathways, including *ceaS2*, *pah2*, *cas2*, *pcbAB*, *pcbC*, and *cefD–cefEF*, thereby linking the production of the β-lactamase inhibitor and its companion antibiotic. Consistent with this finding, lat expression was significantly reduced in *S. clavuligerus*/pIBLD. The Lat enzyme mediates the transformation of lysine into precursors of the cephamycin C pathway [36,37]; thus, its diminished expression could partially explain the observed downregulation of this biosynthetic cluster. Considering that both cephamycin and CA gene sets are activated by the regulator CcaR, the simultaneous downregulation of *lat* and *ccaR* offers a plausible explanation for the coordinated decrease in β-lactam production [38]. Although a GC-rich palindromic motif was observed upstream of *lat*, enrichment and motif-scan analyses did not provide statistical support to assign this sequence as a direct BldD binding site. Thus, the repression of *lat* and *ccaR* in the *bldD*-overexpressing strain is consistent with either direct or indirect regulation, and direct binding will require experimental validation.

BldD may also influence CA production through interactions with upstream regulators of *ccaR*, such as AdpA [39,40]. Although we did not observe an effect on *adpA* expression under our conditions, previous studies in industrial strains reported that *adpA* can be targeted by BldD, correlating with decreased *ccaR* expression [2,4]. Thus, the combined repression of *lat* and downregulation of *ccaR* support a model in which BldD integrates developmental regulation with secondary metabolism, shifting resources away from β-lactam biosynthesis toward morphological differentiation and alternative defense strategies.

Interestingly, downregulation of β-lactamase inhibitors (*blip*) and β-lactamase-related genes suggests that BldD indirectly alters self-resistance mechanisms. One possibility is that altered peptidoglycan recycling reduces signals that normally activate β-lactamase expression, analogous to AmpR-mediated regulation in Gram-negative bacteria [41,42]. While speculative, this could explain the coupled downregulation of cephamycin C, CA, and β-lactamase genes.

These findings reveal that BldD integrates diverse cellular processes by repressing CA and cephamycin C biosynthesis, while promoting developmental genes, terpene biosynthesis, and phage tail-like particles. Importantly, the link with GlcNAc metabolism provides a new perspective on how cell wall recycling and carbon flux might serve as metabolic cues for developmental and ecological strategies in *S. clavuligerus*. Finally, we placed our findings in comparative context using a public *S. coelicolor* bldD-null transcriptome. Cross-species ortholog analyses showed null strict DEG overlap and a biologically negligible global rank-based association, consistent with substantial species-/condition-specific rewiring of BldD-associated outputs. Notably, a clearer and directionally consistent signal emerged within GlcNAc/amino-sugar–related orthologs, supporting the idea that BldD perturbation preferentially impacts amino-sugar/cell-wall recycling modules, whereas downstream effects on specialized metabolism (including β-lactam pathways) are strongly context dependent.

## Conclusions

In *S. clavuligerus*, *bldD* overexpression is associated with transcriptional changes linked to morphological development and specialized metabolism, including increased expression of genes related to phage tail-like particles and terpene biosynthesis. Our results suggest that BldD may help coordinate development with defense and metabolic functions, potentially involving accessory pathways encoded in distinct gene clusters. Overexpression of *bldD* enhanced morphological differentiation, upregulating *bldN*, *bldM*, and chaplin genes, but reduced clavulanic acid and cephamycin C production, consistent with changes in the expression of key regulators including *ccaR*. These findings contrast with the reported repressive role of BldD in *S. coelicolor*, consistent with species- and context-specific regulatory outputs. Overall, BldD appears to shift metabolic priorities toward development at the expense of β-lactam production, warranting further experimental validation.

In summary, BldD functions as a global regulator in *S. clavuligerus*, and its overexpression is consistent with a redistribution of transcriptional programs from β-lactam production toward morphological differentiation and ecological/defense-associated functions. The coordinated changes observed across CA/cephamycin pathways, CIS genes, terpene-related genes, and GlcNAc/amino-sugar metabolism underscore BldD as a central node linking growth, survival, and specialized metabolism.

## Supporting information

S1 File Materials and some representative results on transcriptome analysis. This supplementary material includes: S1 Table: List of bacterial strains used in this study, S2 Table: List of plasmids; S3 Table: Primers used during this study with the restriction sites underlined; S4 Table: Phage-related genes and their potential organization in *S. clavuligerus*; S5 Table: promoter motif logo identified by MEME suite (*E*-value: 8.9e^-28^) in a putative promoter region of 22 up-regulated genes showing affected transcription in *S. clavuligerus*/pIBLD; S1 Fig. Proportion of DEGs annotated in KEGG for *S. clavuligerus*/pIBLD at 72 hours of cultivation. A) Up-regulated. B) Down-regulated; S2 Fig. For upregulated genes, A) TOMTOM analysis showed similarity of the query motif to NagR/NagQ-binding sites (p = 6.38e^-03^). (B) MEME identified a 41-bp AT-rich motif (E = 8.9e^-28^; consensus 5´-RAWNGRARASCATGRNMATKYSVWSAVWDWDYYHKACRAAR-3´), with the conserved core CTCT highlighted by a box. For downregulated genes, C) MEME analysis revealed a conserved GC-rich palindromic motif (E = 4.7e^-22^; consensus 5´-TGCTCCGTCRNNNTCG-3´).
(DOCX)

S2 File Some representative DEGs at 72h. This supplementary material includes: S6 Table: Some representative upregulated genes; S7 Table: Some representative downregulated genes.
(PDF)

S3 File Down and Up-regulated genes at 72h. (XLSX)

S4 File Down and Up-regulated genes at 24h. (XLSX)

S5 File Motif found in the promoter region of the DGE genes. (XLSX)

S6 File FIMO results. (CSV)

S7 File Motif enrichment summary. (CSV)

S8 File Top_GlcNAc_orthologs. (XLSX)

S9 File Top _CIS_phage_orthologs. (XLSX)
