# Supplementary material for: Beyond Clavulanic Acid biosynthesis: Exploring the broad regulatory impact of BldD in Streptomyces clavuligerus ATCC 27064
Source: PLoS One. 2026 Apr 22;21(4):e0347564. doi: 10.1371/journal.pone.0347564 (PMC13102240; doi:10.1371/journal.pone.0347564)
Supplement: S1 File — This supplementary material includes: S1 Table: List of bacterial strains used in this study, S2 Table: List of plasmids; S3 Table: Primers used during this study with the restriction sites underlined; S4 Table: Phage-related genes and their potential organization in S. clavuligerus; S5 Table: promoter motif logo identified by MEME suite (E-value: 8.9e-28) in a putative promoter region of 22 up-regulated genes showing affected transcription in S. clavuligerus/pIBLD; S1 Fig. Proportion of DEGs annotated in KEGG for S. clavuligerus/pIBLD at 72 hours of cultivation. A) Up-regulated. B) Down-regulated; S2 Fig. For upregulated genes, A) TOMTOM analysis showed similarity of the query motif to NagR/NagQ-binding sites (p = 6.38e-03). (B) MEME identified a 41-bp AT-rich motif (E = 8.9e-28; consensus 5´-RAWNGRARASCATGRNMATKYSVWSAVWDWDYYHKACRAAR-3´), with the conserved core CTCT highlighted by a box. For downregulated genes, C) MEME analysis revealed a conserved GC-rich palindromic motif (E = 4.7e-22; consensus 5´-TGCTCCGTCRNNNTCG-3´). (DOCX) [file pone.0347564.s001.docx]

**S1 File:** **Materials and some representative results on transcriptome analysis**

**Materials**

**Bacterial strain**

S1 Table presents the list of bacterial strains used in this study along with a brief description.

**S1 Table.** List of bacterial strains used in this study.

| **Bacteria** | **Description** | **Source or reference** |
| --- | --- | --- |
| *Escherichia coli* DH5α (1) | *F-*ϕ*80lacZΔM15 Δ(lacZYA- argF) U169 recA1 endA1 hsdR17 (rk -, mk +) phoA supE44* λ*- thi-1 gyrA96 relA1* |  |
|  |  | General culture collection Bioprocess group at University of Antioquia  (Colombia) |
| *Escherichia coli* DSM11539  (Migula 1985) ^(2)^ | GM48Sm^r^ (*thr leu thi lacY galK galT ara tonA tsx supE44)*, *DeltalacproF1 traD36 proAB*  *lacIQZDeltaM15*, lambda^-.^ Note: the strain has lost its  dam and dcm markers. |  |
|  |  | DSMZ  German collection of microorganisms |
| *Streptomyces clavuligerus*  ATCC 27064 | Wild type strain, clavulanic acid | Kindly provided by the institute INBIOTEC at Leon University (Spain). |

| *S. clavuligerus*/pIB139 | pIB139 without gene insert integrated into *S. clavuligerus* ATCC27064 (control strain). | This study |
| --- | --- | --- |
| *S. clavuligerus/*pIBLD | *bldD* overexpressed in the wild type *S. clavuligerus*. | This study |
| *S. clavuligerus*/pBLD_1 | *bldD* disrupted in the wild type *S. clavuligerus*. | This study |

- 1. Due to its high transformation efficiency this strain has been used in the transformation and amplification of plasmid DNA.
  2. It has a modification in the methylation system, so that, it is not able to methylate the exogenous DNA that is introduced to it.

# Cloning vectors

S2 Table presents the plasmids used in this study and a brief description of their main characteristics.

**S2 Table.** List of plasmids

| **Plasmid** | | **Characteristic** | **Source or reference** | |
| --- | --- | --- | --- | --- |
| pIB139 | | *int ^ФC31^, att^ФC31^*, *oriT, ermE*,* Aprm^R^ |  | |
| TOPO-TA | | Amp^R^, Neo^R^, pUC ori, *lac*Z | Thermo Fisher | |
| pIBLD | pIB139 with *S. clavuligerus bldD* gene at its *Xba*I-*Nde*I recognition site. | | | This study |

**Composition and culture media**

| **Tryptic Soy Broth (TSB)** | **g/L** |
| --- | --- |
| Tryptic Soy Broth | 30 |
| For solid medium, agar was added at 15 g/L |  |

| **Luria Broth (LB)** | **g/L** |
| --- | --- |
| NaCl | 10 |
| Peptone | 10 |
| Yeast extract | 5 |
| For solid medium, agar was added at 15 g/L. | |

| **Yeast extract-malt extract (YEMEG)** | **g/L** |
| --- | --- |
| Yeast extract | 3 |
| Malt extract | 3 |
| Peptone | 5 |
| Glycerol | 10 |
| Sucrose | 150 |

The pH was adjusted to 7,0. After sterilization for 20 min at 121°C, the following sterile components were added to 100 mL of the culture medium.

| MgCl2 (2,5 M) | 0,2 mL |
| --- | --- |
| Glycine (20%) | 2,5 mL |

# R2YEG protoplast regeneration medium.

Modification of R2YE medium, where glucose has been replaced by glycerol.

| Component | g/L |
| --- | --- |
| Sucrose | 103 |
| K2SO4 | 0,25 |
| MgCl2.6H2O | 10,12 |
| Glycerol | 10 |
| Casamino acids | 0,1 |
| Yeast extract | 5 |

| Trace element solution* | 0,002 |
| --- | --- |

For solid medium, agar was added at 2,2%

After sterilization during 30 min at 121°C, the following sterile components were added to 80 mL of medium:

| Component | mL |
| --- | --- |
| KH2PO4 (0,5%)  CaCl2 (0,25 M) | 1  8 |
| L-proline (20%)  TES buffer (5,73% pH 7,2) | 1,5  10 |
| NaOH (1M) | 0,5 |

Each of the components mentioned above must be autoclaved separately.

| Trace element solution * | **mg/L** |
| --- | --- |
| ZnCl2 | 40 |
| FeCl3. 6H2O | 200 |
| CuCl2·2H2O | 10 |

| **GYM** | **g/L** |
| --- | --- |
| Glucose | 4,0 |
| Yeast extract | 4,0 |
| Malt extract | 10 |
| CaCO3 | 2,0 |
| Agar | 15 |
| Adjust pH to 7.3 before adding agar. |  |

| **Seed medium** | **g/L** |
| --- | --- |
| Glycerol | 15 |
| Peptone | 10 |
| Malt extract | 1,0 |
| MgSO4 | 0.75 |
| MnCl2 | 0.0001 |
| FeSO4 | 0.001 |

| ZnSO4 | 0,001 |
| --- | --- |
| MOPS | 21 |
| K2HPO4 | 0.8 |

The pH was adjusted to 6,8.

| **Soy protein isolate medium (ISP)** | **g/L** |
| --- | --- |
| Glycerol | 30 |
| Soy protein isolate | 25 |
| K2HPO4 | 0.8 |
| MgSO4.7H2O | 0.75 |
| MnCl2·4H2O | 0.0001 |
| FeSO4.7H2O | 0.001 |
| ZnSO4.7H2O | 0.001 |
| MOPS | 21 |

After completely dissolving the components, the pH was adjusted to 6.8. Subsequently, the medium was sterilized for 20 minutes at 121 ° C.

Note: The pre-culture medium has the same composition as ISP, except for glycerol concentration which was adjusted to 15 g/L.

# Oligonucleotides (primers)

**S3 Table.** Primers used during this study with the restriction sites underlined.

| **Primers** | **Sequence (5’→3’)** | **Restriction**  **site** | **Description** |
| --- | --- | --- | --- |
| *bldD*_Fw | ccc**catatg**atgtccagcgaatacgcaaaac | *Nde*I | PCR amplification |
| *bldD*_Rv | ccc**tctaga**tcagctctcctcgtggctgac | *Xba*I |  |

| aac (3) IV_Fw | Cagcttctcaaccttggggt | **-** | To confirm insertion of pIB139 in *S.*  *clavuligerus.* |
| --- | --- | --- | --- |
| aac (3) IV_Rv | Atgatctgctctgcctgtgg | **-** |  |
| ermE*_Fw | Atgctagtcgcggttgatcg |  | To confirm by  sequencing, *bldD*  insertion in pIB139 |

**Transcriptome analysis**

**Proportion of DEGs annotated in KEGG for *S. clavuligerus*/Pibld**


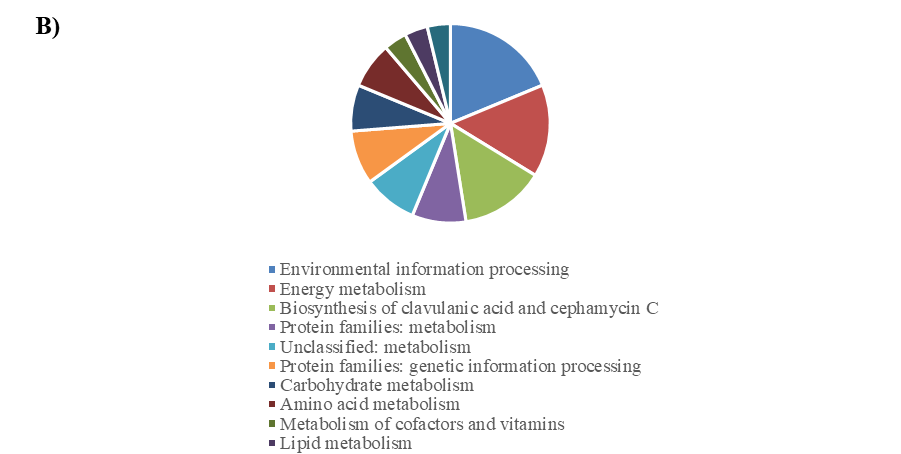

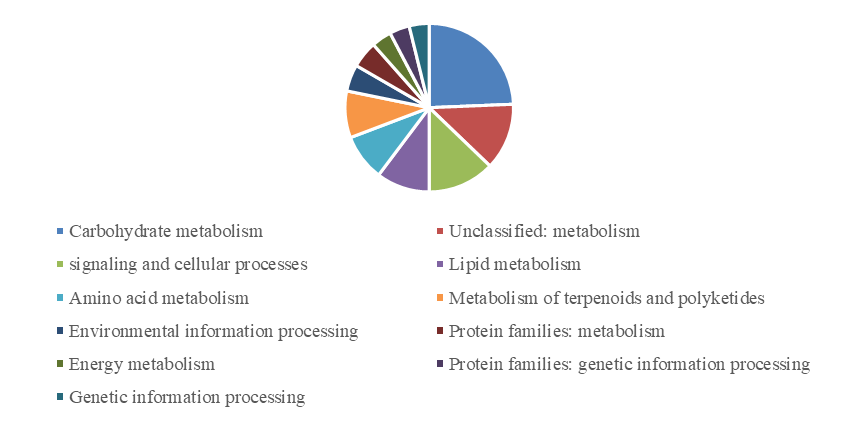


**A)**

**S1 Fig.** Proportion of DEGs annotated in KEGG for *S. clavuligerus*/pIBLD at 72 hours of cultivation. A) Up-regulated; B) Down-regulated

**Organization of phage tail related genes in *Streptomyces clavuligerus***

**S4 Table.** Phage-related genes and their potential organization in *S. clavuligerus*

| **Gene** | **Fold change** | **Gene product** | **Localization** |
| --- | --- | --- | --- |
| CRV15_RS27855 | 3.0 | ATP-binding protein | Chromosome |
| CRV15_RS27860 | 3.0 | Hypothetical protein |  |
| CRV15_RS27865 | 3.0 | Hypothetical protein |  |
| CRV15_RS27870 | 3.0 | Hypothetical protein |  |
| CRV15_RS27875 | 2.5 | Hypothetical protein |  |
| CRV15_RS27880 | 2.9 | Hypothetical protein |  |
| CRV15_RS27885 | 3.1 | Phage tail protein |  |
| CRV15_RS27890 | 3.0 | Putative baseplate assembly protein |  |
| CRV15_RS27895 | 3.1 | Baseplate J/gp47 family protein |  |
| CRV15_RS27900 | 2.8 | GPW/gp25 family protein |  |
| CRV15_RS27905 | 3.0 | Phage baseplate assembly protein V |  |
| CRV15_RS27910 | 3.2 | Phage late control D family protein |  |
| CRV15_RS27915 | 3.0 | LysM peptidoglycan-binding domain containing protein |  |
| CRV15_RS27925 | 3.5 | Phage tail protein |  |
| CRV15_RS27930 | 3.2 | Phage tail sheath |  |
| CRV15_RS27935 | 3.0 | Phage tail protein |  |
| CRV15_RS27940 | 3.2 | Phage tail sheath |  |
| CRV15_RS27945 | 3.0 | Carboxypeptidase-like regulatory domain-containing protein |  |
| CRV15_RS27950 | 2.7 | DUF4255 domain-containing protein |  |

| CRV15_RS27955 | 2.5 | Hypothetical protein |  |
| --- | --- | --- | --- |
| CRV15_RS27960 | 1.0 | BTAD domain-containing putative transcriptional regulator |  |
| CRV15_RS33920 | - | AAAATPase family protein | Plasmid |
| CRV15_RS33915 | - | Hydrolytic protein |  |
| CRV15_RS33910 | - | Transcriptional regulator, SARP family |  |
| CRV15_RS33905 | 1.8 | Phage tail sheat |  |
| CRV15_RS33900 | 1.9 | Phage tail protein |  |
| CRV15_RS33890 | - | Secreted protein |  |
| CRV15_RS33895 | 1.9 | Hypothetical protein |  |
| CRV15_RS33885 | 1.5 | Hypothetical protein |  |
| CRV15_RS33880 | - | PAT1 domain-containing protein |  |
| CRV15_RS33875 | 1.7 | Phage tail protein |  |
| CRV15_RS33870 | 1.5 | LysM peptidoglycan-binding domain-containing protein |  |
| CRV15_RS33865 | 2.0 | VgrG-related protein |  |
| CRV15_RS33860 | 1.9 | GPW/gp25 family protein |  |
| CRV15_RS33855 | 1.5 | Putative baseplane assembly protein |  |
| CRV15_RS33850 | 1.6 | Phage tail protein |  |
| CRV15_RS33845 | 1.6 | Hypothetical protein |  |

**S5 Table.** Putative binding motif logo identified by MEME suite (*E*-value: 8.9e^-28^) in a putative promoter region of 22 up-regulated genes showing affected transcription in S. clavuligerus/pIBLD.

| **Gene** | ***p*-value** | **Site** | | |
| --- | --- | --- | --- | --- |
| CRV15_RS19005 | 3.60e-12 | TTCTCCGGTG | ATTTCACGGCCATGGCAATCCCCTGAATATGTCATACAATC | CGACGTGATG |
| CRV15_RS02805 | 1.81e-11 | TGTCGCCCTG | AAATGGACAACTTGGCAAGGGCCACTCTGAATTTTTCAGAG | TGGGCATATT |
| CRV15_RS02800 | 1.81e-11 | TGTCGCCCTG | AAATGGACAACTTGGCAAGGGCCACTCTGAATTTTTCAGAG | TGGGCATATT |
| CRV15_RS15800 | 1.77e-10 | AAGCAGTAAT | GGTGCGAACTCATTGACGCTTGATCGATGATCTATATAAAC | GATCT |
| CRV15_RS01220 | 1.95e-10 | CCTGTGCCGA | GCCAGAAAATCATGCGCCTGTGTACAGAGTCAACGAAGGAA | GTGTTTCTGA |
| CRV15_RS01215 | 1.95e-10 | CCTGTGCCGA | GCCAGAAAATCATGCGCCTGTGTACAGAGTCAACGAAGGAA | GTGTTTCTGA |
| CRV15_RS20620 | 5.36e-10 | CGTACAGCCG | GAACGTTGCCCATGGTAACGAGAACTGATATCAGTTCTGAA | AGACTGTCAG |
| CRV15_RS22800 | 7.01e-10 | CCATGAATGT | GAATGGTGTCCATGGACATGAGCTGAAACAGCAAGGAGACG | CATCGT |
| CRV15_RS19075 | 8.36e-10 | ACGAACGTCA | GAGTGAATACAATCTGAATCTGGTGAGAAGGCTTGGCGAAG | CGCCTTGCAT |
| CRV15_RS22300 | 1.82e-9 | GGCGGTATCG | GATGAGTCCCCAATAACCTTTGGTGAAAAGGATATTCATGG | ATATCGACCA |
| CRV15_RS09035 | 2.54e-9 | GGTGCGCGA | GAAGGGTAAGGTCGCGAATTTTCTCGAAAATCTTTGCGATC | GACATCCCGC |
| CRV15_RS27960 | 3.83e-9 | GCCCCATTTT | ACGGACAACCCATGATCGTGTGGTGAAAGTCTTCGCATAAA | GGGCGTTTCT |
| CRV15_RS12245 | 6.72e-9 | AGGCGAGTGC | TAATCATTGGCGTTAGCACTCTCCCAGTGAGAGTGACAAAA | GAAGGACCGG |
| CRV15_RS25060 | 1.57e-8 | CAGTCACCGC | AGTAGGAGTCCTTGGGCACGCGCGGACTGTATCCGATGAGG | TGGGGAGGAG |
| CRV15_RS10950 | 1.82e-8 | GATGCAACCG | AGAAAGAAAACTTACACTTTCGGACAAATCTTGCCATATTT | CAATCACTCA |
| CRV15_RS26560 | 4.06e-8 | GAGTTCTGTG | GAGGCTCGGACATGACTCTTCCATGACTTGTTCCGTCGTGA | TTCAGCCATG |
| CRV15_RS08510 | 8.15e-8 | TTTATCCGGG | TCTGATAATGCGACGCATTGCCAACTCTTTACCTTGATGCA | CCGCAACTTC |
| CRV15_RS19005 | 3.60e-12 | TTCTCCGGTG | ATTTCACGGCCATGGCAATCCCCTGAATATGTCATACAATC | CGACGTGATG |
| CRV15_RS02805 | 1.81e-11 | TGTCGCCCTG | AAATGGACAACTTGGCAAGGGCCACTCTGAATTTTTCAGAG | TGGGCATATT |
| CRV15_RS02800 | 1.81e-11 | TGTCGCCCTG | AAATGGACAACTTGGCAAGGGCCACTCTGAATTTTTCAGAG | TGGGCATATT |
| CRV15_RS15800 | 1.77e-10 | AAGCAGTAAT | GGTGCGAACTCATTGACGCTTGATCGATGATCTATATAAAC | GATCT |
| CRV15_RS01220 | 1.95e-10 | CCTGTGCCGA | GCCAGAAAATCATGCGCCTGTGTACAGAGTCAACGAAGGAA | GTGTTTCTGA |
| CRV15_RS01215 | 1.95e-10 | CCTGTGCCGA | GCCAGAAAATCATGCGCCTGTGTACAGAGTCAACGAAGGAA | GTGTTTCTGA |
| CRV15_RS20620 | 5.36e-10 | CGTACAGCCG | GAACGTTGCCCATGGTAACGAGAACTGATATCAGTTCTGAA | AGACTGTCAG |
| CRV15_RS22800 | 7.01e-10 | CCATGAATGT | GAATGGTGTCCATGGACATGAGCTGAAACAGCAAGGAGACG | CATCGT |
| CRV15_RS19075 | 8.36e-10 | ACGAACGTCA | GAGTGAATACAATCTGAATCTGGTGAGAAGGCTTGGCGAAG | CGCCTTGCAT |
| CRV15_RS22300 | 1.82e-9 | GGCGGTATCG | GATGAGTCCCCAATAACCTTTGGTGAAAAGGATATTCATGG | ATATCGACCA |
| CRV15_RS09035 | 2.54e-9 | GGTGCGCGA | GAAGGGTAAGGTCGCGAATTTTCTCGAAAATCTTTGCGATC | GACATCCCGC |
| CRV15_RS27960 | 3.83e-9 | GCCCCATTTT | ACGGACAACCCATGATCGTGTGGTGAAAGTCTTCGCATAAA | GGGCGTTTCT |
| CRV15_RS12245 | 6.72e-9 | AGGCGAGTGC | TAATCATTGGCGTTAGCACTCTCCCAGTGAGAGTGACAAAA | GAAGGACCGG |
| CRV15_RS25060 | 1.57e-8 | CAGTCACCGC | AGTAGGAGTCCTTGGGCACGCGCGGACTGTATCCGATGAGG | TGGGGAGGAG |
| CRV15_RS10950 | 1.82e-8 | GATGCAACCG | AGAAAGAAAACTTACACTTTCGGACAAATCTTGCCATATTT | CAATCACTCA |
| CRV15_RS26560 | 4.06e-8 | GAGTTCTGTG | GAGGCTCGGACATGACTCTTCCATGACTTGTTCCGTCGTGA | TTCAGCCATG |
| CRV15_RS08510 | 8.15e-8 | TTTATCCGGG | TCTGATAATGCGACGCATTGCCAACTCTTTACCTTGATGCA | CCGCAACTTC |
| **Motif Consensus** | | | RAWNGRARASCATGRNMATKYSVWSAVWDWYYHKACRAAR |  |

**Promoter motif enrichment analysis**

**A**


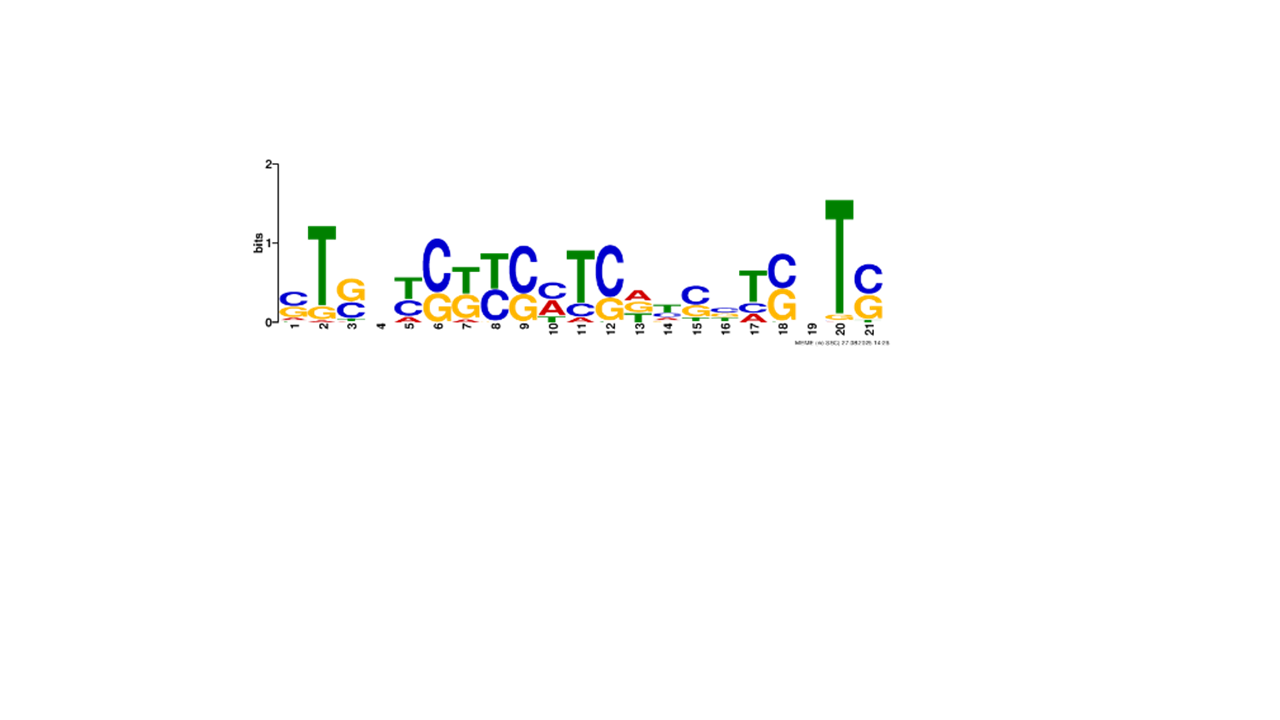

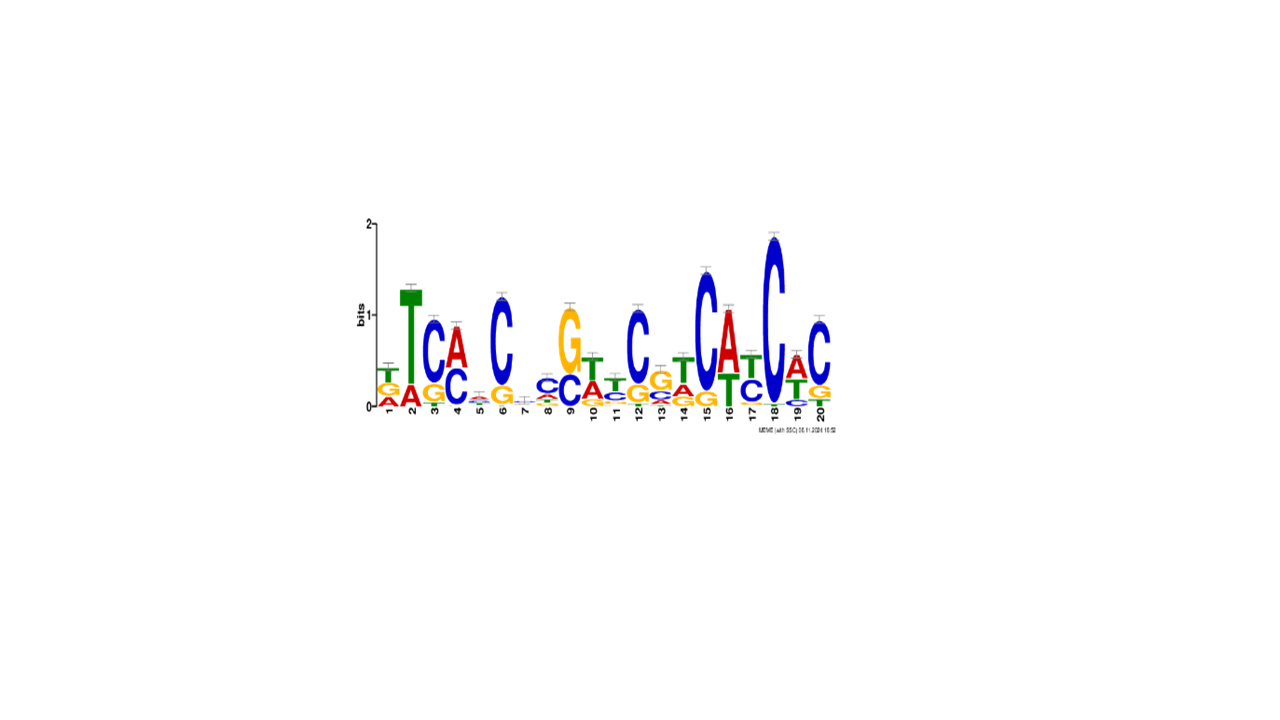

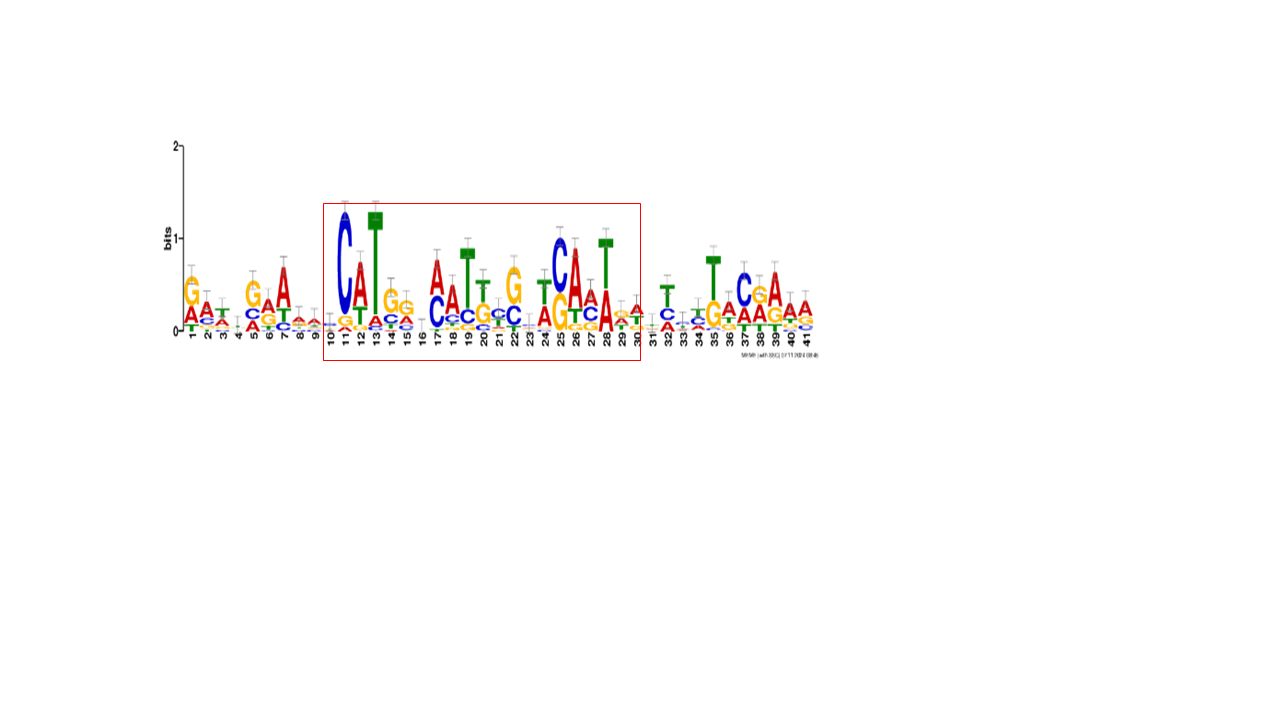


**B**

**C**

**S2 Fig**. For upregulated genes, A) TOMTOM analysis showed similarity of the query motif to NagR/NagQ-binding sites (p = 6.38e-03). (B) MEME identified a 41-bp AT-rich motif

(E = 8.9e-28; consensus 5´-RAWNGRARASCATGRNMATKYSVWSAVWDWDYYHKACRAAR-3´), with the conserved core CTCT highlighted by a box. For downregulated genes, C) MEME analysis revealed a conserved GC-rich palindromic motif (E = 4.7e-22; consensus 5´-TGCTCCGTCRNNNTCG-3´).
