# Supplementary material for: Beyond Clavulanic Acid biosynthesis: Exploring the broad regulatory impact of BldD in Streptomyces clavuligerus ATCC 27064
Source: PLoS One. 2026 Apr 22;21(4):e0347564. doi: 10.1371/journal.pone.0347564 (PMC13102240; doi:10.1371/journal.pone.0347564)
Supplement: S2 File — This supplementary material includes: S6 Table: Some representative upregulated genes; S7 Table: Some representative downregulated genes. (PDF) [file pone.0347564.s002.pdf]

Some representative DEGs at 72 h

S6 Table. Some representative upregulated genes

**Carbohydrate metabolism**				
**Genes**	**Protein name**	**log2FoldChange**	**padj**	**Reaction or metabolic process**
CRV15_RS20620	Aldehyde dehydrogenase	1.5	$7.7x10^{-5}$	glycolysis/gluconeogenesis
CRV15_RS32220	Glyoxalase superfamily protein	4.5	$1.1x10^{-32}$	Glyoxylate and dicarboxylate metabolism
CRV15_RS26985	PTS fructose transporter	1.3	$5.5x10^{-3}$	glycolysis/gluconeogenesis
CRV15_RS12090	N-acetylmuramic acid 6-phosphate etherase (murQ)	1.6	$1.1x10^{-3}$	Sugar metabolism
CRV15_RS27375	N-acetylmuramoyl-L-alanine amidase	1.6	$7.2x10^{-6}$	Sugar metabolism
**Lipid metabolism**				
CRV15_RS25055	Lipid-transfer protein	2.0	$6.2x10^{-6}$	Lipid biosynthesis
CRV15_RS25615	Lysophospholipid acyltransferase family protein	1.5	$1.5x10^{-4}$	Phospholipids biosynthesis
CRV15_RS27320	acyl-CoA synthetase	1.1	$3.2x10^{-2}$	Fatty acid biosynthesis
CRV15_RS28650	acyl-CoA desaturase	3.5	$1.7x10^{-4}$	Fatty acid metabolism
**Metabolism of terpenoids and polyketides**				
CRV15_RS00240	Polyprenyl synthetase family protein	2.3	$2.5x10^{-3}$	Terpenoid backbone biosynthesis
CRV15_RS02380	shc squalene hopene cyclase	1.2	$2.7x10^{-2}$	Sesquiterpenoid and triterpenoid biosynthesis
CRV15_RS27370	Terpene synthase family protein	1.5	$1.4x10^{-5}$	Sesquiterpenoid and triterpenoid biosynthesis
CRV15_RS31155	Terpene synthase	1.3	$9.2x10^{-3}$	Terpenoid backbone biosynthesis

CRV15_RS11485	Acyclic terpene utilization protein	1.5	$1.2x10^{-2}$	Terpenoid backbone biosynthesis
**Transcriptional regulators**				
CRV15_RS27945	Carboxypeptidase-like regulatory domain-containing protein	2.9	$1.1x10^{-14}$	unknown
CRV15_RS37415	LuxR family transcriptional regulator	2.2	$9.8x10^{-5}$	unknown
CRV15_RS09965	Response regulator transcription factor (*bld*M)	2.3	$6.1x10^{-9}$	Morphological differentiation
CRV15_RS15315	XRE family transcriptional regulator	2.1	$3.2x10^{-6}$	unknown
**Miscellaneous genes**				
CRV15_RS09035	Vps62-related protein	5.3	$7.7x10^{-39}$	Unknown
CRV15_RS03785	SapB/AmfS family lanthipeptide	3.7	$6.0x10^{-6}$	Lanthipeptid biosynthesis
CRV15_RS03790	SapB/AmfS family lanthipeptide	3.7	$2.8x10^{-18}$	Lanthipeptid biosynthesis
CRV15_RS26965	nagB amine-6-phosphate deaminase	1.3	$4.3x10^{-3}$	Aminosugars metabolism
CRV15_RS16730	sigma-70 family RNA polymerase sigma factor (*bld*N)	1.4	$1.9x10^{-3}$	Morphological differentiation
CRV15_RS27195	SigB/SigF/SigG family RNA polymerase sigma factor	2.2	$4.9x10^{-5}$	Morphological differentiation
CRV15_RS23175	Chaplin	2.1	$1.2x10^{-4}$	Morphological differentiation

Some representative downregulated genes

S7 Table. Some representative downregulated genes

Energy metabolism				
**Genes**	**Protein name**	**log2FoldChange**	**padj**	**Reaction or metabolic process**
CRV15_RS04045	Nitrite/sulfite reductase	-3.2	$6.5x10^{-14}$	Sulfur metabolism
CRV15_RS04060	Adenylyl-sulfate kinase	-3.3	$1.2x10^{-15}$	Sulfur metabolism
CRV15_RS04070	Sulfate adenylyltransferase subunit 1	-3.0	$2.99x10^{-11}$	Sulfur metabolism
CRV15_RS04065	Sulfate adenylyltransferase subunit CysD	-2.5	$1.1x10^{-9}$	Sulfur metabolism
CRV15_RS12515	sulfurtransferase	-3.1	$3.6x10^{-12}$	Sulfur metabolism
**Biosynthesis of amino acids**				
CRV15_RS33280	Bifunctional ornithine acetyltransferase/N-acetylglutamate synthase	-4.5	$4.2x10^{-2}$	Arginine biosynthesis
CRV15_RS32010	Isoprenylcysteine carboxylmethyltransferase	-4.7	$2.4x10^{-2}$	Methyltransferase activity
CRV15_RS13875	Homoserine O-acetyltransferase/O-succinyltransferase	-2.9	$2.0x10^{-3}$	Biosynthesis of methionine and sulfur metabolism
**Miscellaneous genes**				
CRV15_RS32005	AfsA-related hotdog domain-containing protein	-4.9	$2.5x10^{-2}$	Unknown
CRV15_RS20155	Serine hydrolase domain-containing	-3.8	$1.1x10^{-6}$	NA
CRV15_RS11635	Peptidoglycan-binding domain-containing	-2.4	$1.9x10^{-6}$	NA
CRV15_RS32355	Serine hydrolase domain-containing	-2.0	$1.3x10^{-6}$	NA
CRV15_RS04920	BLIP family protein	-1.6	$7.9x10^{-5}$	NA
